# Trends and racial disparity in primary pressure ulcer hospitalizations outcomes in the US from 2005 to 2014

**DOI:** 10.1097/MD.0000000000035307

**Published:** 2023-10-06

**Authors:** Shahrzad Bazargan-Hejazi, Marvin Ambriz, Shakir Ullah, Shahbaz Khan, Maria Bangash, Kaveh Dehghan, Chizobam Ani

**Affiliations:** a Department of Psychiatry, College of Medicine, Charles R. Drew University of Medicine and Science and UCLA David Geffen School of Medicine, Los Angeles, CA, USA; b College of Medicine, Charles R. Drew University of Medicine and Science and UCLA David Geffen School of Medicine, Los Angeles, CA, USA; c Department of Internal Medicine, College of Medicine, Charles R. Drew University of Medicine and Science, Los Angeles, CA, USA; d Ayub Medical College, Pakistan; e Southern California University of Health and Sciences, CA, USA; f College of Medicine, Charles R. Drew University of Medicine and Science and UCLA David Geffen School of Medicine, Los Angeles, CA, USA; g College of Medicine, Charles R. Drew University of Medicine and Science and UCLA David Geffen School of Medicine, Los Angeles, CA, USA.

**Keywords:** pressure ulcer inflation-adjusted charges, pressure ulcer length of hospital stay, pressure ulcer mortality, primary pressure ulcer disparity, race-ethnicity

## Abstract

In the United States (US), pressure ulcers affect ≤3 million people and costs exceed 26.8 billion US dollars in spending. To examine trends in primary pressure ulcer (PPU) hospitalization mortality, length of hospital stay (LOS), and inflation-adjusted charges (IAC) in the US from 2005 to 2014 by race/ethnicity. We secondarily examined the relationship between race/ethnicity with PPU mortality, LOS, and IAC with race/ethnicity. This cross-sectional study used Nationwide Inpatient Sample (NIS) data from 2005 to 2014. The study sample included all hospitalizations with the designated ICD-9-CM code of 707.20-25 (pressure ulcer). There was a notable decline in PPU hospitalization from 11.5% to 7.77 % between 2005 and 2014. The mean mortality decreased from 2.32% to 1.12% (*P* < .001), the mean LOS declined from 9.39 days (*P* < .001), and the mean IAC per hospitalization decreased from $30,935 to $29,432 (*P* < .001). Positive changes observed in mortality, LOS, and IAC trends were consistent across different racial and ethnic groups. The results of multivariable logistic and linear regression analyses revealed that Black patients (β = 0.68, 95% CI 0.36–1.01, *P* < .001) and patients belonging to the Other race/ethnic category (β = 0.93, 95% CI 0.18–1.69) had longer hospital stays compared to their White counterparts. Regarding IAC, Black patients (β = 2846, 95% CI 1254–4439, *P* < .005), Hispanic patients (β = 6527, 95% CI 4925–8130), and patients from the Other race/ethnic category (β = 3473, 95% CI 1771–5174) had higher IAC for PPU treatment compared to their White counterparts. PPU hospitalization discharges, as well as hospitalization mortality, LOS, and IAC, decreased during the study period, however, our findings revealed disparities in PPU outcomes among different racial/ethnic groups. Implications of the findings are discussed.

## 1. Introduction

A pressure ulcer (PU) or pressure injury, as defined by the National Pressure Ulcer Advisory Panel, refers to an area of localized skin and underlying tissue damage caused by excessive and prolonged pressure, shear stress, or friction.^[1,2]^ This condition is associated with increased pain, risks of infection, healthcare costs, reduced quality of life for patients,^[3]^ and even mortality.^[4–6]^ Annually, pressure ulcers affect approximately 3 million people in the United States,^[7]^ resulting in healthcare expenditures of around $26.8 billion.^[8]^

Hospital-acquired pressure ulcers is costly, reaching up to $70,000 per patient due to high treatment expenses and extended hospital stays.^[9]^ In 2008, Medicare implemented a hospital-acquired conditions policy to penalize hospitals providing poor quality care, aiming to reduce preventable complications such as primary pressure ulcers (PPU).^[10]^ While some studies showed a decline in hospital-acquired pressure ulcers following the policy implementation, the rates remained relatively stable in subsequent years.^[11,12]^

Despite an overall decrease in PPU rates, racial disparities have persisted over time, with Hispanic and Black individuals being disproportionately affected.^[5,13]^ For example, studies have shown a higher incidence of PPU in Black patients compared to non-Hispanic White patients in nursing homes.^[14,15]^ However, there is a lack of reporting on race/ethnicity disparities in PPU hospitalization rates, mortality, length of stay, and inflation-adjusted charges (IAC), using national inpatient data, using a national sample.^[16]^

This study aims to examine trends in PPU hospitalization mortality, length of hospital stay (LOS), and IAC in the United States from 2005 to 2014, focusing on race/ethnicity. The study also explores the relationship between race/ethnicity and PPU mortality, LOS, and IAC. The findings may inform interventions and resource allocation to address disparities in minority patients’ presentation of this condition.

## 2. Methods

### 2.1. Design, setting, and participants

This cross-sectional study utilized data from the Nationwide Inpatient Sample (NIS) from 2005 to 2014. NIS is a component of the Healthcare Cost and Utilization Project, a collaborative partnership between the Federal government, State agencies, and the healthcare industry, sponsored by the Agency for Healthcare Research and Quality (AHRQ).^[17]^ It captures discharge-level information on primary and secondary diagnoses and procedures, discharges vital statuses, and demographics on discharges per year.^[18]^ The unit of analysis for the current study was hospitalization discharges. The study sample included all hospitalizations with the designated ICD-9-CM code of 707.20-25 (pressure ulcer), which has been a common approach in previous studies.^[18,19]^ The analytic weighted sample comprised 474,332 hospitalizations with a diagnosis of PPU.

### 2.2. Measures

We assessed several key outcomes, including a) PPU hospitalizations, b) in-hospital mortality, c) LOS, and d) IAC (indexed to 1997 US mean hospitalization costs).^[20]^ The study main independent variable was race/ethnicity, categorized as White, Black, Hispanic, and Other race/ethnicity. To account for potential confounding variables, covariates encompass socio-demographic variables such as age, gender, insurance status, and income, as well as hospital bed sizes and medical co-morbidity burdens, measured using Charlson Comorbidity Index (CCI).^[21]^ The study adhered to ethical guidelines, and the university ethics committee waived the need for review since the dataset used was publicly available and de-identified.

### 2.3. Data analysis

We assessed all study variables for normality, and for those that were non-normal, they were appropriately categorized. Following the requirements of the NIS, we post-stratified our data using the relevant NIS weight, cluster, and stratum variables. Subsequently, we examined the distribution of the study variables and explored crude trends in each study outcome from 2005 to 2014. These trends were further analyzed by race/ethnicity. Regression trend lines were computed for each outcome, and the R2 trends were presented. we conducted bivariate analyses using chi-square tests and 1-way analysis of variance to examine the distribution of the study outcomes and covariates by race/ethnicity.

For assessing the independent relationship between race/ethnicity and mortality, we employed multivariable logistic regression analysis, presenting odds ratios and 95% confidence intervals. Additionally, we used multivariable linear regression analyses (Table 4) to examine the relationship between race/ethnicity and LOS and IAC, displaying the regression coefficient and its corresponding 95% confidence interval.

Throughout the analysis, we thoroughly examined the underlying assumptions for all regression analyses and made appropriate adjustments as needed. A significance level of 0.05 was set for all analyses. To conduct these analyses, we utilized SAS version 9.4 ©.

## 3. Results

As depicted in Table 1, PPU hospitalizations declined from 11.55% to 7.77% between 2005 and 2014. Among the hospitalization cases, 64.6% were White patients, 21.3% followed by 21.3% and 9.62% Black and Hispanic patients, respectively. Additionally, 51.6% of the cases involved patients aged 65 or older. The majority of hospitalizations (51.61%) were for individuals aged 65 years and older, with males comprising 51.18% of the sample. Notably, Medicare users accounted for slightly over 66% of the hospitalization cases. Over the study period from 2005 to 2014, the average hospital mortality rate for PPU was 1.75% (n = 9797). The mean LOS was 8.52 days (S.E 0.09), and the mean IAC per hospitalization amounted to $30,360 (S.E $457.12).

**Table 1:** Characteristics of the discharges with diagnosis of pressure ulcer (N = 1,401,838).

Variable	Pressure ulcer diagnosis	
	Percentage/mean (SE)	Weighted n
Age 18–44 45–64 65–74 ≥75	16.34% 32.05% 15.38% 36.23%	89,274 175,084 83,989 197,905
Gender Female	48.82%	279,824
Race/ethnicity Whites Blacks Hispanic Others*	64.56% 21.31% 9.62% 4.52%	306,219 101,075 45,617 21,422
Insurance status Medicare Medicaid Private insurance Others†	65.94% 14.40% 13.59% 6.07%	369,780 80,773 76,235 34,031
Income for zip code $1–$38,999 $39,000–$47,999 $48,000–$62,999 ≥ $63,000	35.31% 25.65% 21.67% 17.37%	193,105 140,275 118,501 949,957
Hospital bed-size Small Medium Large	15.18% 26.30% 58.52%	84,938 147,029 327,191
CCI	2.00 (0.01)	116,307
Mortality	1.75%	9797
LOS (days)	8.52 (0.09)	116,307
IAC	$30,360 (457.12)	114,891
Yr 2005 2006 2007 2008 2009 2010 2011 2012 2013 2014	11.55% 12.03% 11.12% 10.92% 10.65% 9.66% 9.50% 8.80% 8.01% 7.77%	64,876 67,557 62,480 61,325 59,850 54,257 53,353 49,405 45,020 43,630

CCI = Charlson Comorbidity Index, IAC = inflation adjusted charges, LOS = length of hospital stay.

* Asian or Pacific Islander, Native American, Other.

† Self-pay, no charge, others missing, invalid, and unavailable.

**Figure 1.**
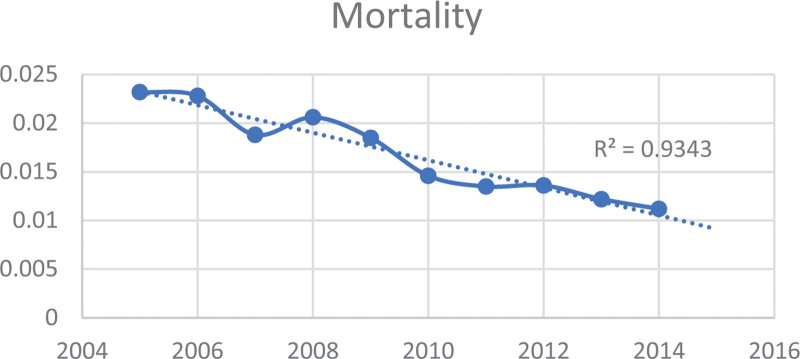
Trend in mortality rate (per 100,000) for primary pressure ulcer hospitalization from 2005 to 2014.

**Figure 2.**
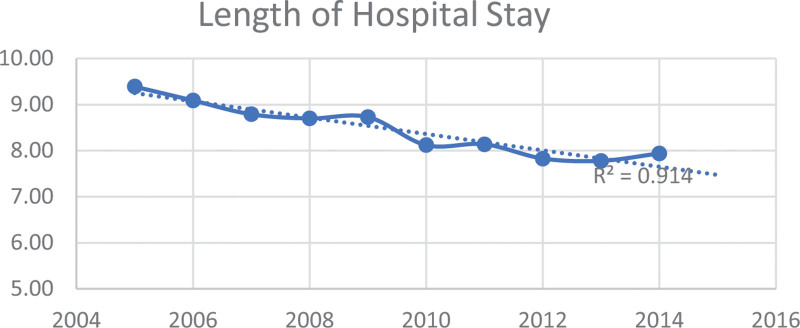
Trend in mean length of stay for primary pressure ulcer hospitalization from 2005 to 2014.

**Figure 3.**
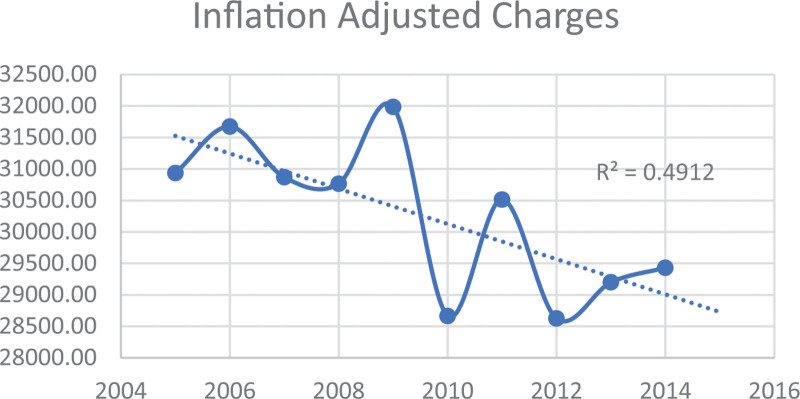
Trend in inflation-adjusted charges for primary pressure ulcer hospitalization from 2005 to 2014.

Table 2 and Figures 1 to 3 display the trends in mortality, LOS), and IAC for PPU diagnosis from 2005 to 2014 in the study sample. The data illustrates a downward trend in these outcomes over the study period by race/ethnicity. Specifically, the mean mortality decreased from 2.32% to 1.12% (*P* < .001). Furthermore, the mean LOS declined from 9.39 days (SE = 0.27) to 7.94 days (SE = 0.15) (*P* < .001). Additionally, the mean IAC per hospitalization decreased from $30,935 (SE = $1087) to $29,432 (SE = $697) (*P* < .001).

**Table 2:** Trends in pressure ulcer mortality, LOS, and inflation adjusted charges (IAC) in general.

Variables	2005	2006	2007	2008	2009	2010	2011	2012	2013	2014	Total	Sig.
	%(SE)/Mean (SE)	%(SE)/Mean (SE)	%(SE)/Mean (SE)	%(SE)/Mean (SE)	%(SE)/Mean (SE)	%(SE)/Mean (SE)	%(SE)/Mean (SE)	%(SE)/Mean (SE)	%(SE)/Mean (SE)	%(SE)/Mean (SE)	%(SE)/Mean (SE)	
Mortality	2.32%	2.28%	1.88%	2.06%	1.85%	1.46%	1.35%	1.36%	1.22%	1.12%	1.75%	<0.001
LOS	9.39 (0.27)	9.09 (0.23)	8.79 (0.23)	8.70 (0.23)	8.73 (0.28)	8.12 (0.25)	8.14 (0.27)	7.83 (0.13)	7.78 (0.14)	7.94 (0.15)	8.52 (0.09)	<0.001
IAC	$30,935 (1087)	$31,674 (1402)	30,870 (1038)	$30,767 (1028)	$31,984 (1414)	$28,668 (1052)	30,514 (1479)	$28,625 (642)	$29,200 (624)	$29,432 (697)	$30,360 (455)	<0.001

IAC = inflation adjusted charges, LOS = length of hospital stay.

Table 3 presents the unadjusted association between race/ethnicity and other covariates with mortality, LOS, and IAC in patients with PPU. While there was no significant association between mortality and race/ethnicity, we found a significant relationship between LOS and race/ethnicity (*P* < .001). On average, Black patients and patients from the Other race/ethnicity category had longer hospital stays, at 9.35 ± 0.17 and 9.38 ± 0.37 days, respectively, followed by Hispanic patients at 8.56 ± 0.19 days.

**Table 3:** Characteristics of the discharges with diagnosis of pressure ulcer by race and ethnicity (N = 474,332).

Variable	White		Blacks		Hispanic		Other		Sig. % (SE)	Total	
	Weighted frequency	% (SE)	Weighted frequency	% (SE)	Weighted frequency	% (SE)	Weighted frequency<?Char=Unit?>	Weighted frequency		Weighted frequency	% (SE)
Age											
18–44	39,965	52.95 (0.87)	22,617	29.96 (0.78)	9093	12.05 (0.56)	3807	5.04 (0.25)		75,482	
45–64	94,575	64.16 (0.70)	32,523	22.07 (0.53)	13,694	9.29 (0.45)	6626	4.50 (0.20)	<.0001	147,419	
65–74	47,385	66.65 (0.70)	13,861	19.50 (0.54)	6660	9.37 (0.58)	3189	4.48 (0.27)		71,095	
≥75	116,203	69.38 (0.93)	28,993	17.31 (0.52)	15,027	8.97 (0.97)	7272	4.34 (0.28)		167,495	
Total										461,491	100 (N/A)
Gender											
Female	151,510	63.38 (0.70)	51,990	21.75 (0.52)	24,580	10.28 (0.51)	10,969	4.59 (0.20)		239,050	
Male = 1	154,673	65.75 (0.72)	49,075	20.86 (0.53)	21,037	8.94 (0.66)	10,452	4.44 (0.21)	<.0001	235,237	
Total										474,287	100 (N/A)
Insurance Status											
Medicare	213,465	68.35 (0.70)	60,025	19.22 (0.49)	25,916	8.30 (0.65)	12,900	4.13 (0.21)		312,305	
Medicaid	30,920	44.60 (0.95)	23,954	34.55 (0.85)	10,512	15.16 (0.87)	3953	5.70 (0.30)		69,340	
Private insurance	44,755	71.43 (0.65)	10,529	16.80 (0.54)	4531	7.24 (0.40)	2836	4.52 (0.26)	<.0001	62,652	
Others*	16,575	56.68 (1.30)	6337	21.67 (0.80)	4621	15.80 (0.94)	1711	5.85 (0.43)		29,244	
Total										473,542	100 (N/A)
Income/Zip Code											
$1–$38,999	81,889	49.91 (0.91)	54,120	32.99 (0.85)	21,122	12.88 (0.84)	6933	4.23 (0.23)		164,065	
$39,000–$47,999	80,458	70.27 (0.72)	19,967	17.44 (0.55)	9733	8.50 (0.47)	4332	3.79 (0.22)	<.0001	114,491	
$48,000–$62,999	71,520	72.15 (0.93)	14,344	14.47 (0.52)	8698	8.78 (0.85)	4574	4.61 (0.35)		99,135	
≥$63,000	65,577	78.18 (0.67)	9615	11.47 (0.52)	4411	5.26 (0.35)	4269	5.09 (0.34)		83,871	
Total										461,562	100 (N/A)
Hospital Bed-size											
Small	50,270	69.29 (1.14)	13,263	18.28 (1.03)	5050	6.70 (0.54)	3971	5.48 (0.45)		72,553	
Medium	80,546	63.04 (1.45)	27,007	21.14 (0.89)	14,095	11.03 (1.59)	6131	4.80 (0.37)	0.0012	127,780	
Large	173,930	64.01 (0.90)	60,239	22.18 (0.72)	26,382	9.71 (0.62)	11,150	4.10 (0.25)		271,701	
Total										472,033	100 (N/A)
Yr											
2005	33,094	68.23 (1.51)	8260	17.03 (1.01)	5297	10.92 (1.42)	1848	3.81 (0.44)		48,500	
2006	32,877	63.87 (1.85)	11,596	22.53 (1.55)	5173	10.05 (1.25)	1826	3.54 (0.40)		51,472	
2007	29,429	63.60 (1.95)	10,153	21.94 (1.60)	4529	9.79 (1.48)	2156	4.66 (0.55)		46,268	
2008	31,829	65.82 (1.51)	9966	20.61 (1.30)	4040	8.35 (0.88)	2518	5.20 (0.68)		48,353	
2009	32,773	63.68 (1.78)	10,356	20.12 (1.30)	5238	10.17 (1.52)	3096	6.01 (0.73)	0.0639	51,462	
2010	30,486	62.85 (1.68)	11,464	20.63 (1.35)	4487	9.25 (1.29)	2069	4.26 (0.43)		48,506	
2011	31,346	64.25 (1.45)	10,950	22.44 (1.21)	4449	9.12 (1.15)	2043	4.19 (0.42)		48,787	
2012	30,440	65.12 (0.82)	9895	21.16 (0.66)	4330	9.26 (0.59)	2086	4.45 (0.31)		46,745	
2013	27,490	64.45 (0.83)	9305	21.81 (0.70)	4030	9.45 (0.54)	1825	4.28 (0.27)		42,650	
2014	26,455	63.60 (0.82)	9130	21.95 (0.71)	4045	9.72 (0.53)	1960	4.71 (0.30)		41,590	
Total										474,332	100 (N/A)
Mortality											
No	300,415	64.53 (0.67)	99,276	21.33 (0.50)	44,843	9.63 (0.56)	20,988	4.50 (0.18)			
Yes	5441	65.41 (1.44)	1728	20.78 (1.22)	733	8.81 (0.98)	416	5.00 (0.66)	0.5551		
Total										473,840	100 (N/A)

	Mean (SE)	Weighted Frequency	Mean (SE)	Weighted Frequency	Mean (SE)	Weighted Frequency	Mean (SE)	Weighted Frequency		Mean (SE)	Weighted Frequency
LOS (days)	8.43 (0.10)	63,354	9.35 (0.17)	20,896	8.56 (0.19)	9459	9.38 (0.37)	4387	<0.001	8.52 (0.09)	116,307
IAC	$29.910 (461.3)	62,844	$34,592 (1041.9)	20,578	$35,871 (1107.9)	9334	$35,210 (1325.5)	4325	<0.001	$30,360 (455.5)	114,891
CCI	1.95 (0.01)	63,364	2.19 (0.02)	20,901	2.04 (0.05)	9463	2.04 (0.03)	4388	<0.001	2.00 (0.01)	116,327

CCI = Charlson Comorbidity Index, IAC = inflation adjusted charges, LOS = length of hospital stay.

*Self-pay.

Regarding IAC, our study revealed a significant correlation between the charges incurred in the treatment of PPU and race/ethnicity (*P* < .001). Hispanic patients had the highest average IAC at $35,871 ± 1107.9, followed by patients from the Other race/ethnicity category at $35,210 ± 1325.5, and Black patients at $34,592 ± 1041.9. In contrast, White patients spent the least on PPU with an average IAC of $29,910 ± 461.3.

Table 4 presents the results of multivariable logistic and linear regression analyses examining the association of PPU-related mortality, LOS, and IAC with race/ethnicity while controlling for potential confounding variables. We did not observe any significant difference in PPU mortality based on race/ethnicity. However, concerning LOS, our findings indicated that Black patients (β = 0.68, 95% CI 0.36–1.01, *P* < .001) and patients belonging to the Other race/ethnic category (β = 0.93, 95% CI 0.18–1.69, *P* < .001) had longer hospital stays compared to their White counterparts. Regarding IAC, our study revealed that Black patients (β = 2846, 95% CI 1254–4439, *P* < .005), Hispanic patients (β = 6527, 95% CI 4925–8130, *P* < .001), and patients from the Other race/ethnic category (β = 3473, 95% CI 1771–5174, *P* < .001) had higher IAC for PPU treatment compared to their White counterparts. Furthermore, we examined the potential interaction between CCI and race/ethnicity for mortality, LOS, and IAC, but no significant interactions were observed (data not shown).

**Table 4:** Multivariate analyses of pressure ulcer mortality, length of hospital stay and inflation adjusted charges by study variables in the US from 2005 to 2014.

Variables	Mortality weighted	Sig.	LOS weighted	Sig.	IAC weighted	Sig.
	n = 445,771		n = 446,144		n = 439,876	
Variable	OR (95% CI)		β (95% CI)		β (95% CI)	
Age 18–44 (ref) 45–64 65–74 ≥75	1.00 (N/A) 3.19 (2.31, 4.40) 6.16 (4.36, 8.70) 11.19 (8.00, 15.66)	N/A 0.003 <0.0001 <0.0001	N/A −0.38 (−0.66, −0.10) −0.75 (−1.11, −0.40) −1.16 (−1.50, −0.82)	N/A 0.0084 <0.0001 <0.0001	N/A 1302 (513.2, 2091) 609.7 (−486.3, 1705) −1609 (−2666, −551.5)	N/A 0.0012 0.2756 0.0029
Race/ethnicity Whites (ref) Blacks Hispanic Other*	1.00 (N/A) 1.08 (0.94, 1.25) 0.98 (0.81, 1.18) 1.17 (0.91, 1.50)	N/A 0.70 0.34 0.29	N/A 0.68 (0.36, 1.00) −0.02 (−0.40, 0.35) 0.93 (0.18, 1.69)	N/A <0.0001 0.9117 0.0146	N/A 2846 (1254, 4439) 6527 (4925, 8130) 3473 (1771, 5174)	N/A 0.0005 <0.0001 <0.0001
Gender Male Female	1 (NA) 0.99 (0.89, 1.09)	N/A 0.979	N/A −0.13 (−0.29, 0.02)	0.1026	N/A −436.1 (−915.7, 43.53)	
Insurance status Medicare(ref) Medicaid Private insurance Other†	1.00 (N/A) 1.05 (0.82, 1.35) 1.30 (1.08, 1.55) 1.14 (0.85, 1.55)	N/A 0.51 0.05 0.84	N/A 0.83 (0.43, 1.22) −0.74 (−1.05, −0.44) −0.95 (−1.40, −0.50)	N/A <0.0001 <0.0001 <0.0001	N/A −1001 (−1987, −15.10) 647.3 (−216.3, 1510) −1883 (−3168, −597.7)	N/A 0.0466 0.1418 0.0041
Zip code income $1–$38,999 (ref) $39,000–$47,999 $48,000–$62,999 ≥$63,000	1.00 (N/A) 0.84 (0.73, 0.97) 0.87 (0.75, 1.01) 0.92 (0.79, 1.08)	N/A 0.11 0.43 0.78	2.82 (1.21, 4.44) 2.95 (1.34, 4.56) 3.00 (1.39, 4.61) 3.28 (1.66, 4.90)	0.0006 0.0003 0.0003 <0.0001	2578 (−9489, 14,646) 3937 (−8116, 15,991) 6090 (−5977, 18,158) 9873 (−2234, 21,981)	0.6753 0.5220 0.3225 0.1100
Hospital bed size Small (ref) Medium Large	1.00 (N/A) 0.79 (0.65, 0.95) 0.85 (0.71, 1.01)	N/A 0.02 0.51	N/A −1.03 (−1.90, −0.17) −0.29 (−1.14, 0.55)	N/A 0.0182 0.4967	N/A 4435 (2442, 6428) 7149 (5173, 9125)	N/A <0.0001 <0.0001
CCI Mean (SE)	1.19 (1.16, 1.21)	<.0001	0.39 (0.35, 0.43)	<0.0001	1021 (869.1, 1174.8)	<0.0001

Sample includes participant ≥ ^*β*^Rao Chi square.

CCI = Charlson Comorbidity Index, IAC = inflation adjusted charges, LOS = length of hospital stay.

* Asian or Pacific Islander, Native American, Other.

† Self-pay, no charge, others missing, invalid, and unavailable from source.

## 4. Discussion

**Figure 4.**
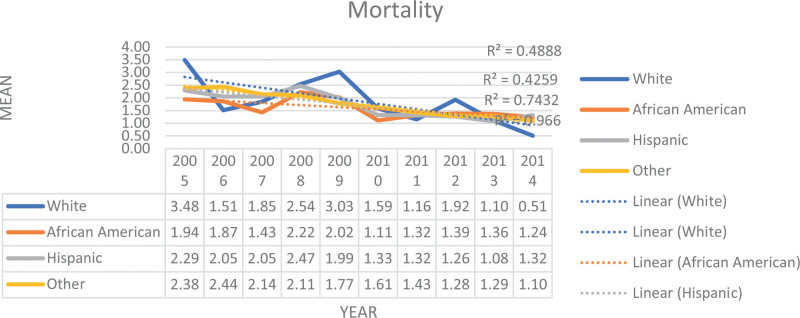
Trend in mortality rate (per 100,000) for primary pressure ulcer hospitalization by race, from 2005 to 2014.

**Figure 5.**
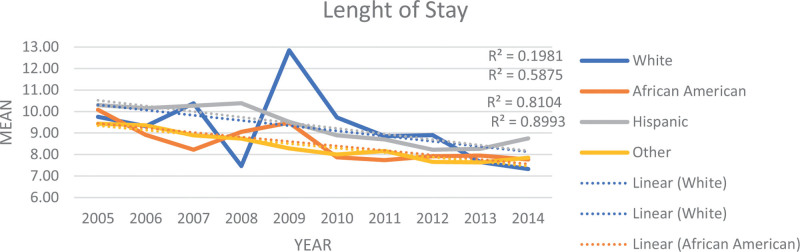
Trend in mean length of stay for primary pressure ulcer hospitalization by race from 2005 to 2014.

**Figure 6.**
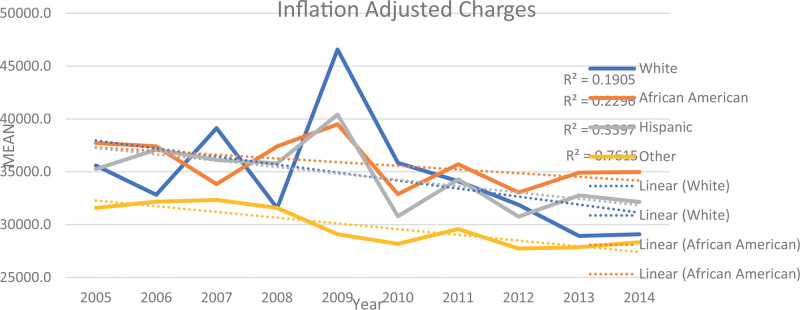
Trend in mean inflation adjusted charges for primary pressure ulcer hospitalization by race from 2005 to 2014.

The study investigated the changes in PPU hospitalization and outcomes, including mortality, LOS, and IAC over a ten-year period. Notably, we observed a significant decline, nearly 4%, in PPU hospitalization (Table 1). The analysis of mortality, LOS, and IAC trends for PPU patients over the study period indicate a positive trend with significant improvements in these outcomes (Table 2, Figures 1–3). These findings indicate improvement in survival rates for patients with PPU and signifying a reduction in the duration of hospitalization for PPU cases, and a decrease in the cost of PPU management during the study period. These trends were consistent across different racial/ethnic groups as shown in Figures 4–6.

The unadjusted association between race/ethnicity and outcomes (Table 3) indicated no significant association between race/ethnicity and mortality. However, race/ethnicity was found to be a significant factor in determining the LOS and IACs. Specifically, disparities were observed for the Black, Hispanics and patients from the Other race/ethnic category. These groups experienced longer hospital stays and higher charges compared to White patients.

Accounting for the confounding variables (Table 4), our study revealed that race/ethnicity remained a significant factor influencing LOS and IAC for PPU treatment. Black, Hispanic, and patients from Other race/ethnicity category continued to experience longer hospital stays and higher charges compared to White patients. However, we observed no significant differences in PPU-related mortality based on race/ethnicity, after controlling for the confounding variables.

The previous retrospective national inpatient data supports the observed overall decline in PPU discharges, mortality, LOS, IAC.^[9,11,16,22]^ This decline partly indicates implementing prevention strategies introduced by Medicare and Medicaid Services encouraging hospitals and nursing homes to draw on best practices to prevent and improve inpatient PPU management has been effective.^[23]^ However, the nursing home studies support the upward PPU trend in Black individuals.^[13]^ Additionally, the literature reflects the disproportionate impact of PPU on LOS and IAC among minority discharges.^[24,25]^

Our findings, along with those of others have important implications for understanding disparities in PPU treatment outcomes based on race/ethnicity. The observed disparities in LOS and IAC highlights the need to identify the specific factors contributing to longer hospital stays and higher charges in minority patients to design effective strategies for equitable health delivery. Potential contributing factors, such as cultural factors, access to healthcare resources, and systemic biases, may influence the differences in PPU outcomes among racial and ethnic groups. Understanding of the role of these factors could help with designing system-level protocols, and policies for PPU care to reduce these disparities. At the patient level, understanding of the unique needs of minority patients could inform healthcare professionals in providing culturally sensitive and inclusive care environment to improve PPU management.

### 4.1. Study limitations

Our study has several limitations that should be considered when interpreting the results. The NIS unit of analysis is the hospitalization event (discharges), not individual patients, so duplicate hospitalizations are treated as independent observations, which may produce some level of bias in the analysis. Second, our findings do not distinguish between patients who required routine preventive care for pressure ulcers and those who presented with a high-risk profile for PPU development. For example, the impact of PPU on patient outcomes could vary depending on the severity of the wound and specific characteristics of the pressure ulcer, as attending to thicker wounds and higher pressure ulcer stages could lead to longer hospital stays and more costly treatments.^[8,26]^ Future research aiming to replicate our findings should consider including patient-level characteristics such as comorbidities, immobility and poor nutrition, as potential risk factors in the analysis.^[27]^

The cross-sectional nature of the study design limits our ability to establish causality between PPU and LOS. That is, it is challenging to determine if PPU caused prolonged hospital stays or vice versa based on this type of analysis. Other studies have associated longer LOS with pressure ulcer development.^[28–30]^ However, longitudinal approach with time series analysis or randomized control trails could better establish causal relationships. Yet, observational study such as the current one analyzing existing data from national databases, offer the advantage of using preexisting data without intervening in patients’ treatment pathway. This approach is more ethical and feasible due to the nature of the PPU and allows us to gain valuable insights into trends and associations.

Furthermore, while we examined the interaction between CCI and race/ethnicity in relation to LOS, and IAC, this interaction did not significantly reduce the observed racial disparities. However, it is important to acknowledge the evidence of racial dipartites in specific health conditions, such as obesity^[31]^ and diabetes,^[32]^ which might warrant further investigation to better understand their potential impact of PPU outcomes.

Nevertheless, it is important to note that this study represents one of few analysis conducted in this area. Additionally, it is the first study to examine NIS data from 2005 to 2014. As such, further research is warranted to validate and expand upon our findings, helping to provide a more robust understanding of racial disparities in PPU management and outcomes.

## 5. Conclusions

From 2005 to 2014, PPU hospitalization discharges as well as hospitalization mortality, LOS, and IAC decreased, indicating positive trends in PPU management. However, our findings revealed disparities in PPU outcomes among different racial/ethnic groups. Specifically Black and patients from Other ethnicities were at a high risk of experiencing PPU, LOS and higher hospitalization costs compared to White patients. Also, Hispanic patients experienced higher expenses for PPU hospitalization than While patients. Notable, race/ethnicity did not emerge as a significant factor influencing hospitalization mortality.

Disparities observed for Black and Hispanic groups warrant further investigation to identify underlying causes, since these disparities have potential implications for healthcare equity and overall well-being of affected individuals. Moreover, to gain a comprehensive understanding of PPU outcomes, future similar studies should distinguish between high-risk profile patients and differentiate between PPU and secondary pressure ulcer hospitalizations outcomes across different race/ethnic groups. Such research will provide valuable insights to devise targeted interventions that could effectively reduce disparities and improve the overall management of PPU for the minority populations.

## Author contributions

**Conceptualization:** Shahrzad Bazargan-Hejazi, Marvin Ambriz, Chizobam Ani.

**Data curation:** Chizobam Ani.

**Formal analysis:** Marvin Ambriz, Kaveh Dehghan, Chizobam Ani.

**Funding acquisition:** Shahrzad Bazargan-Hejazi.

**Methodology:** Chizobam Ani.

**Resources:** Shahbaz Khan, Maria Bangash, Kaveh Dehghan.

**Supervision:** Shahrzad Bazargan-Hejazi, Chizobam Ani.

**Validation:** Shakir Ullah, Shahbaz Khan, Maria Bangash, Kaveh Dehghan, Chizobam Ani.

**Writing – original draft:** Shahrzad Bazargan-Hejazi, Marvin Ambriz.

**Writing – review & editing:** Shahrzad Bazargan-Hejazi, Shakir Ullah, Shahbaz Khan.
